# Evaluation of the efficiency and safety of combined chemotherapy and molecular-targeted therapy in the treatment of advanced gastric cancer: A protocol for systematic review and meta-analysis

**DOI:** 10.1097/MD.0000000000027557

**Published:** 2021-11-12

**Authors:** Zhan He, Jian-Guo Xu

**Affiliations:** a Department of General Surgery, Jinhua People's Hospital, Jinhua, Zhejiang, China; b Department of Radiology, Zhejiang Provincial People's Hospital, Hangzhou, China.

**Keywords:** chemotherapy, gastric cancer, meta, molecular therapy

## Abstract

**Background:**

Gastric cancer is considered to be the sixth prevalent cancer and the third widespread trigger of cancer-associated deaths globally. One of the major method of treating this harmful condition is completely resecting the entire tumor. Standard treatment procedures, including radiotherapy, surgery, and chemotherapy are ineffective for patients with advanced gastric cancer (AGC), mainly because the predictions are deficient. Many studies have recently sought to examine the effect of combining chemotherapy and molecular-targeted therapy, supposing that such developments could become effective for treating AGC. Still, the advantages of combining chemotherapy plus molecular-targeted therapy to treat advanced gastric cancer appear to be unconvincing.

**Methods and analysis:**

We intend to perform an electronic search using information obtained from PubMed, EMBASE, Cochrane Library, ScienceDirect, Web of Science, China National Knowledge Infrastructure, and WanFang databases. Specifically, we will consider all randomized controlled trials published in English or Chinese, and focus only on those assessing the effectiveness and safety of a MIC of chemotherapy and molecular-targeted therapy to treat AGC. Furthermore, two independent authors will conduct data extraction as well as explore the risk of bias. Furthermore, we intend to use the odds ratio for dichotomous data, mean differences or standardized mean differences for continuous data, along with hazard ratio for time-to-event data, with 95% confidence intervals (CIs).

**Ethics and dissemination:**

Because of the nature of this study, we will not require ethical approval. Instead, we will report the review reported in a peer-reviewed journal.

## 1 Introduction

Gastric cancer (GC) is among commonest cancerous tumors and the sixth in terms of its prevalence. It is also the third disease accounting for the highest mortality rate among all types of.^[1]^ In particular, many studies indicate that GC incidences have reduced in many developed countries. Also, some studies consider that the prevalence or incidence rates among men and women have slowed.^[2]^ Until now, the most effective strategy to treat GC has been comprehensive therapy involving surgery.^[3,4]^ However, the characteristic symptoms that patients with GC are identified with, including “advanced-stage tumors, tend to decrease the probability of reaction, resulting in a poor 5-year survival rate”.^[5]^ Specifically, it means that prevalence and progressions of GC “activation of oncogenes and inactivation of tumor suppressor genes”.^[6]^ While the healing impact in GC improves due to the current utilization of targeted medicine advanced on molecular biology research of GC, the 5-year rate of survival seems to have remained constant.^[3,7]^

Chemotherapy enhances patients’ quality of life and extends survival than supportive care alone.^[8,9]^ Therefore using a mix of chemotherapy, by mixing fluorouracil and a platinum compound, as well as adding a third drug (usually docetaxel or epirubicin), has become the typical first-line regimen to treat advanced gastric cancer (AGC).^[10,11]^ Still, the reactions tend to be partial and limited and are considered concerning toxicities.^[9]^ The increasing comprehension of the fundamental molecular basis of carcinogenesis has instigated the development of targeted agents, presenting assuring outcomes to treat patients experiencing lung, colon, breast, or kidney cancers. Even though this might tentatively control the use of molecular-targeted therapy, many clinical trials seem to demonstrate promising effectiveness when a targeted agent (trastuzumab) is added to the typical chemotherapy for HER-2 positive patients with AGC.^[12]^

In contrast, there are no pieces of evidence, including systematic reviews or meta-analyses that critically appraise the possible effectiveness and safety of using combined chemotherapy and molecular-targeted therapy to treat AGC. To this end, the current study will carry out a systematic review of randomized controlled trials (RCTs) to explore the underlying evidence on effectiveness and safety of combined chemotherapy and molecular-targeted therapy for treating AGC.

## 2 Methods and analysis

The meta-analysis’ protocol was registered on OSF (https://osf.io) under the number 10.17605/OSF.IO/6UWHN. Also, it has been written according to the guidelines postulated by the Preferred Reporting Items for Systematic Reviews and Meta-analysis Protocols.^[13]^

## 3 Inclusion criteria for study selection

### 3.1 Types of studies

We aim to incorporate all RCTs. However, the study will exclude any trial with no matching comparison groups.

### 3.2 Types of participants

We intend to also incorporate AGC patients of all ages, mainly those with diagnostic criteria of AGC using histologically confirmed adenocarcinoma.

### 3.3 Types of interventions

We will treat the intervention group with chemotherapy and molecular-targeted therapy and treat the comparison group with conventional chemotherapy alone, molecular-targeted agents alone, or no treatment at all.

### 3.4 Types of outcomes

The anticipated primary outcomes comprise of general survival and development-free survival. The expected secondary outcomes are general response, improved quality of life, and unfavorable incidences or consequences.

## 4 Search methods for identification of studies

### 4.1 Electronic searches

We will perform an electronic search using PubMed, EMBASE, Cochrane Library, ScienceDirect, Web of Science, China National Knowledge Infrastructure, and WanFang databases. Accordingly, we will include all RCTs published in English or Chinese, primarily those that examine the efficacy and safety of using combined chemotherapy and molecular-targeted therapy to treat AGC. We will use the following key search terms: [(“gastric cancer” OR “gastric carcinoma” OR “gastric tumor” OR “gastric tumor” OR “gastric neoplasm”) AND (“chemotherapy” OR “target∗∗” OR)] AND (“randomized controlled trial” OR “randomized clinical trial”).

### 4.2 Searching other resources

We intend to check references lists of all the primary studies and reviewed articles to establish extra references. Furthermore, we intend to contact the authors of recognized trials and request them to help in identifying other published articles.

## 5 Data collection and analysis

### 5.1 Selection of studies

We will use 2 independent reviewers to re-examine and inspect all titles and abstracts and find suitable trials according to the set inclusion criteria. Any discrepancies between the authors will be addressed by discussing. The Preferred Reporting Items for Systematic Reviews and Meta-analysis-compliant flow diagram summarizes details of the selection procedure that was used in this investigation.^[14]^

### 5.2 Data extraction and management

Also, the 2 independent authors utilized a standard data collection form to examine the features and outcome data – which was piloted on at least one of the studies to extract the following information: methods: “study design, setting, study date, withdrawals, total duration study”; patient characteristics: “age, gender, number, the severity of the condition, diagnostic criteria”; intervention: “type of intervention, dose, and schedule”; and outcomes: “primary and secondary outcomes specified and collected, time points reported.” Any discrepancies between the authors will be addressed by discussing.

### 5.3 Assessment of risk of bias in included studies

We will also utilize 2 independent authors to investigate the risk of bias by employing a collaboration tool recommended by the Cochrane Handbook 5.1.^[15]^ We will evaluate aspects such as concealment of allocation random allocation, blinding, selective and incomplete outcomes, as well as other predispositions. Any disagreements between the authors will be addressed by discussion.

### 5.4 Measures of treatment effect

Furthermore, we intend to utilize the odds ratio for dichotomous data, mean differences or standardized mean differences for continuous data, and hazard ratio for time-to-event data, with 95% confidence intervals.

### 5.5 Dealing with missing data

We will make get in touch with investigators and study sponsors to indicate main features of the study.

### 5.6 Assessment of heterogeneity

We will study the reasons for the existence of substantial heterogeneity between different studies from different perspectives. Where necessary, we will adopt sensitivity analysis or subgroup analysis to explain the heterogeneity.

### 5.7 Assessment of reporting biases

We will utilize funnel plots to establish possible reporting bias where more than 10 studies are included. We will employ Egger test to establish the asymmetry of the funnel plots.

### 5.8 Assessment of reporting biases

Preferably, sensitivity analyses will be carried out to authenticate the robustness of the inferences. We intend to also assess the effects of sample size, study design, methodological quality, and missing data. Lastly, the analysis will be repeated by eliminating reviews that have low methodological quality.

## 6 Discussion

This review sought to evaluate the efficiency and safety of combined chemotherapy and molecular-targeted therapy to treat AGC. We suppose that the results of the review will focus on addressing the existing gap in the literature. From our standpoint, no study has previously considered a combination of chemotherapy and molecular-targeted therapy to treat AGC. To this end, using systematic review and meta-analysis will be crucial in evaluating the efficiency and safety of chemotherapy combined with molecular-targeted therapy for treating AGC. Our review anticipates providing a basis for chemotherapy plus molecular-targeted therapy for treating patients with AGC and provide a better option to treat such patients.

## Author contributions

**Conceptualization:** Zhan He, Jian-Guo Xu.

**Data curation:** Zhan He, Jian-Guo Xu.

**Formal analysis:** Zhan He, Jian-Guo Xu.

**Funding acquisition:** Jian-Guo Xu.

**Investigation:** Zhan He.

**Methodology:** Zhan He.

**Project administration:** Zhan He, Jian-Guo Xu.

**Resources:** Zhan He, Jian-Guo Xu.

**Software:** Zhan He.

**Supervision:** Jian-Guo Xu.

**Validation:** Zhan He, Jian-Guo Xu.

**Visualization:** Zhan He, Jian-Guo Xu.

**Writing – original draft:** Zhan He, Jian-Guo Xu.

**Writing – review & editing:** Jian-Guo Xu.
